# Gut microbiome comparability of fresh-frozen versus stabilized-frozen samples from hospitalized patients using 16S rRNA gene and shotgun metagenomic sequencing

**DOI:** 10.1038/s41598-019-49956-7

**Published:** 2019-09-16

**Authors:** Emma E. Ilett, Mette Jørgensen, Marc Noguera-Julian, Gedske Daugaard, Daniel D. Murray, Marie Helleberg, Roger Paredes, Jens Lundgren, Henrik Sengeløv, Cameron MacPherson

**Affiliations:** 1 PERSIMUNE Centre of Excellence, Rigshospitalet, Blegdamsvej 9, DK-2100, Copenhagen Ø, Denmark; 2 Institut de Recerca de la SIDA – IrsiCaixa, Hospital Universitari Germans Trias i Pujol, Badalona, Catalonia Spain; 3 Department of Oncology, Rigshospitalet Blegdamsvej 9, DK-2100, Copenhagen Ø, Denmark; 4 Infectious Diseases Service, Hospital Universitari Germans Trias i Pujol, Badalona, Spain; 5 Department of Haematology, Rigshospitalet, Blegdamsvej 9, DK-2100 Copenhagen Ø, Denmark

**Keywords:** Metagenomics, Haematological cancer, Medical research

## Abstract

Collection of faecal samples for microbiome analysis in acutely sick patients is logistically difficult, particularly if immediate freezing is required (i.e. fresh-frozen, or *FF* sampling). Previous studies in healthy/non-hospitalized volunteers have shown that chemical stabilization (i.e. stabilized-frozen, or *SF* sampling) allows room-temperature storage with comparable results to *FF* samples. To test this in a hospital setting we compared *FF* and *SF* approaches across 17 patients undergoing haematopoietic stem cell transplantation (HSCT) using both 16S rRNA gene and shotgun metagenomic sequencing. A paired (same stool specimen) comparison of *FF* and *SF* samples was made, with an overall comparable level in relative taxonomic abundances between the two sampling techniques. Though shotgun metagenomic sequencing found significant differences for certain bacterial genera (*P* < 0.001), these were considered minor methodological effects. Within-sample diversity of either method was not significantly different (Shannon diversity *P*_*16SrRNA*_ = 0.68 and *P*_*shotgun*_ = 0.89) and we could not reject the null hypothesis that between-sample variation in *FF* and *SF* were equivalent (*P*_*16SrRNA*_ = 0.98 and *P*_*shotgun*_ = 1.0). This indicates that *SF* samples can be used to reliably study the microbiome in acutely sick patient populations, thus creating and enabling further outcomes-based metagenomic studies on similarly valuable cohorts.

## Introduction

It is becoming increasingly clear that analysis of the human gut microbiome can reveal critical aspects of human disease pathogenesis, as well as the variation of treatment responses among a variety of patient groups^1–3^. As a result, a growing number of hospitals are trying to establish clinical biobanks of stool samples for use in downstream research projects.

Currently, the considered “gold standard” for microbiome studies suggests that stool samples be collected and then undergo immediate DNA-extraction or at least immediate freezing at −20/−80 °C. However, it is very difficult to comply with these conditions in a hospital setting, where even refrigeration of samples is difficult due to limited resources, irregular sampling time-points or patient visits, as well as the need to prioritize patient care. Additionally, complete capture and follow-up of many patient populations would require at-home sampling of out-patients, adding further variability to the time between sampling and freezing. Therefore, the ideal sample collection method within hospitals would rather allow for samples to be kept at room temperature prior to freezing. This would enable at-home sample collection for out-patients, no need for urgent hospital carriers for sample delivery, and no requirement for same-day DNA-extraction.

Although chemical stabilization followed by a delayed time to freezing (stabilized-frozen freeze (stable-freeze, or *SF*) appears to be a good alternative to direct freezing (fresh-freeze, or *FF*) or fresh DNA-extraction (fresh-extract, or *FE*), all studies to date assessing its use are based on cohorts of non-hospitalized/healthy volunteers^4–22^. In particular, there is an open question as to the use of *SF* protocols when microbiomes are expected to have low diversity^8^. It is therefore currently uncertain whether or not results from previous method studies can be applied to samples collected in a hospital, where low diversity gut microbiomes are likely. Additionally, there have been little data evaluating the effect of *SF* methods using shotgun metagenomic sequencing^4,7,14^, which, unlike 16S rRNA gene sequencing data, provides detailed species level information. As bacterial species can differ in diseased populations, it is essential to collect this information and evaluate whether *SF* affects their compositions or not.

To address these remaining aspects of collection-associated bias within gut microbiomes of the diseased, we have conducted a study comparing *FF* samples to those collected using a *SF* protocol. This is the first study (to our knowledge) assessing the differences between these protocols using both 16S rRNA gene and shotgun metagenomic sequencing in a hospital setting. Our study was performed in a cohort of patients undergoing haematopoietic stem cell transplantation (HSCT) at Rigshospitalet, University of Copenhagen, Denmark. Samples were delivered to the Centre of Excellence for Personalised Medicine for Infectious Complications in Immune Deficiency (PERSIMUNE)^23^ biobank, also located at Rigshospitalet. HSCT serves as a useful disease proxy as it is reserved for severely diseased patients and, critically, has been shown to heavily impact microbiome structure^24^.

## Results

**Figure 1:**
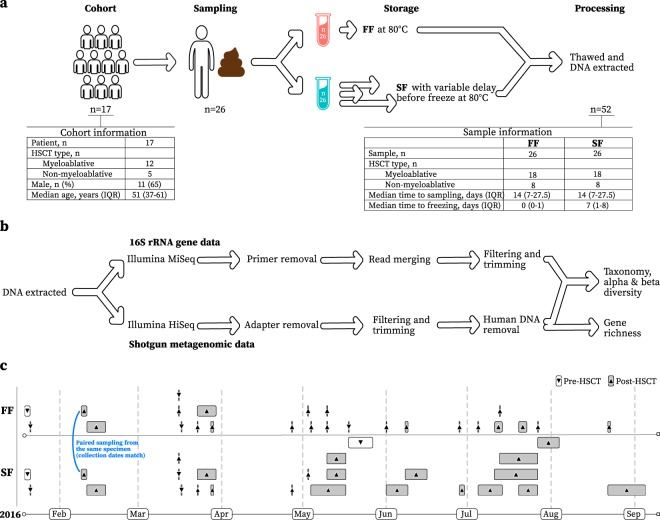
(**a**) Overview of study design with general patient and sample information. FF = Fresh-frozen, SF = Stabilized-frozen. (**b**) Overview of the bioinformatic pipeline. (**c**) Overview of calendar time from sample collection to freezing for all FF (fresh-frozen) and SF (stabilized-frozen) samples. Arrows indicate if pre-HSCT or post-HSCT samples.

A total of 26 paired stool samples were generated from 17 haematology patients undergoing myeloablative or non-myeloablative HSCT. Each stool sample pair consisted of 1 *FF* sample (i.e. freshly frozen at time of sampling), and 1 *SF* sample (i.e. chemically stabilized prior to freezing after a variable period no greater than 16 days). An overview of the study design, summarized baseline patient and sample information, as well as time from sampling to freezing can be found in Fig. 1. Specific patient/sample numbers with time from HSCT to sampling and freezing can be found in Supplementary Table S1.

### Data quality

To ensure that data from both sampling methods were acceptable for analysis, all samples underwent quality control steps as described in the methods section.

#### 16S rRNA gene sequencing data

One sample was removed due to a severe lack of overlap between paired-ends (a fundamental requirement of our analysis pipeline). Another sample was lost due to low sequence content (<10,000 unfiltered reads). We observed a minor batch effect across sample runs that slightly biased mean quality and read numbers by affecting reverse-end sequences. However, our pipeline largely removed this bias by merging paired ends and filtering (see Methods). All filtered samples had mean quality scores of over 34 with >10,000 reads and batch effects did not influence which samples passed quality control steps. To summarize, high quality data was collected for 24 of the 26 stool samples.

#### Shotgun metagenomic sequencing data

Seven samples were removed as their sequenced data did not contain sufficient information for further study, either due to poor quality, human contamination, or sequencing issues (threshold of 1 million reads). All samples had final mean quality scores of over 37 with > 3.5 million reads. To summarize, high quality data was collected for 21 of the 26 stool samples, resulting in a paired sample overlap of 76% (i.e. 76% of paired samples had both 16S rRNA gene and shotgun metagenomic sequencing data after quality control steps). The other 24% of samples had either 16S rRNA gene or shotgun metagenomic sequencing data available for analysis.

### Alpha-diversity

We next assessed possible differences between methods for within-sample diversity (i.e. alpha-diversity).

#### 16S rRNA gene sequencing data

**Figure 2:**
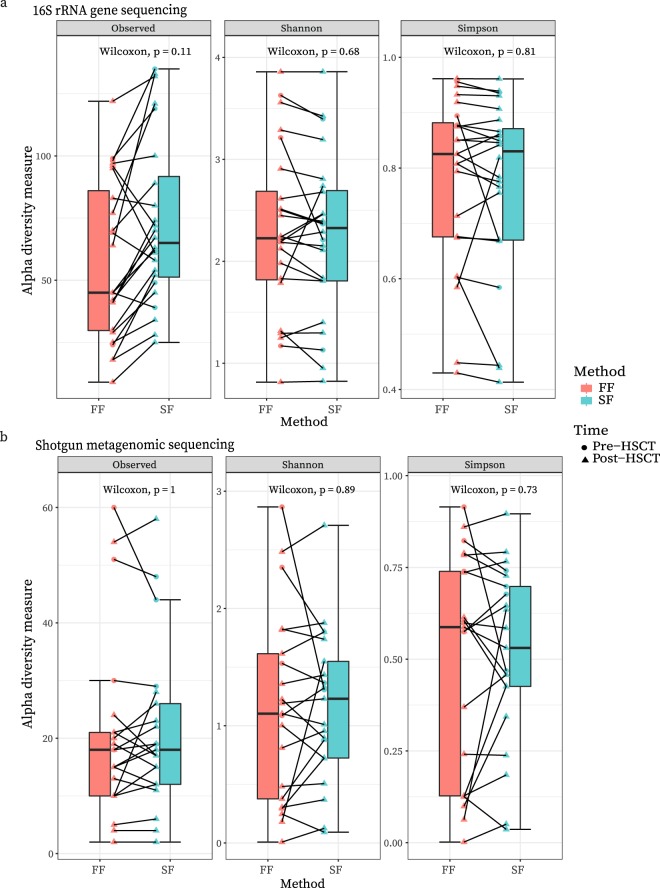
(**a**) Alpha diversity measures based on 16S rRNA gene data for each sampling method, with pairs connected by lines. Circle vs triangle shapes represent pre-HSCT vs post-HSCT samples respectively. There was no statistically significant difference between fresh-frozen (FF) and stabilized-frozen (SF) samples regarding the number of observed species, Shannon or Simpson indexes. (**b**) Alpha diversity measures based on shotgun metagenomic data for each sampling method, with pairs connected by lines. Circle vs triangle shapes represent pre-HSCT vs post-HSCT samples respectively. There was no statistically significant difference between fresh-frozen (FF) and stabilized-frozen (SF) samples regarding the number of observed species, Shannon or Simpson indexes. These figures have been created with help of the R(v3.5.0)^29^/PhyloSeq^37^ package.

We found a general increase of observed genera in the *SF* versus *FF* samples. However, this was not significant (Wilcoxon paired test, *P* = 0.11) and other alpha diversity measures showed no differences (Fig. 2a). The mean Simpson index was 0.78 (IQR 0.68-0.88) vs 0.77 (IQR 0.67-0.87) in *FF* and *SF* samples respectively. Similarly, the mean Shannon indices were likewise comparable at 2.30 (IQR 1.82-2.69) vs 2.25 (IQR 1.80-2.69) in *FF* and *SF* samples respectively. Indeed, there was no statistically significant difference in either alpha diversity measure (Wilcoxon paired test, *P*_Simpson_ = 0.81 and *P*_Shannon_ = 0.68).

#### Shotgun metagenomic sequencing data

As with 16S rRNA gene sequencing, the mean Simpson and Shannon indices were similar between *FF* and *SF* samples at 0.50 (IQR 0.13-0.74) vs 0.51 (IQR 0.43-0.70) and 1.15 (IQR 0.38-1.61) vs 1.16 (IQR 0.72-1.55), respectively (Fig. 2b). As expected from the IQRs there was no significant difference in either diversity measure (Wilcoxon paired test, *P*_Simpson_ = 0.73 and *P*_Shannon_ = 0.89).

#### Shotgun metagenomics gene richness

As an additional measure to assess diversity we also examined gene richness (the number of unique genes) in the shotgun metagenomic sequencing data. This measure is used to divide samples into those of low versus high gene richness, with those of high gene richness generally considered to have higher diversity.

**Figure 3:**
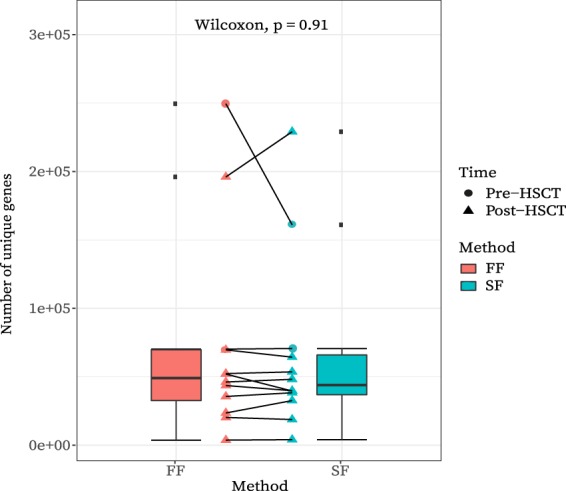
Gene richness (i.e. the number of unique genes) for each sample per method and connected as sample pairs. Circle vs triangle shapes represent pre-HSCT vs post-HSCT samples respectively. Box plots show interquartile ranges (minimum and maximum values as whiskers) for gene richness per sampling method. A Wilcoxon paired test found no significant difference in the mean gene richness between fresh-frozen (FF) and stabilized-frozen (SF) samples. This figure has been created with help of the R(v3.5.0)^29^/PhyloSeq^37^ package.

There was a median of 5,507,430 reads per sample mapping to the IGC (Integrated Gene Catalogue)^25^ (interquartile range (IQR) 4,460,836–6,047,576). Based on a rarefaction curve of mapped reads and the number of unique genes (Supplementary Fig. S1), samples with <5 million mapped reads were discarded for the gene richness analysis as their diversity was biased by sampling. The 12 remaining sample pairs had a median of 47,180 unique genes per sample (IQR 34,876–69,799). *FF* and *SF* samples had similar medians of 49,086 and 44,004, respectively (Fig. 3). There was no evidence of a statistically significant difference in gene richness between *FF* and *SF* samples (Wilcoxon paired test, *P* = 0.91). Two paired samples are notable for their unusually high gene richness (Fig. 3**)**.

### Beta-diversity

We next assessed the beta-diversity, or between-sample diversity, looking for changes in variance between paired samples and testing whether potential differences were driven by sampling method.

#### 16S rRNA gene sequencing data

**Figure 4:**
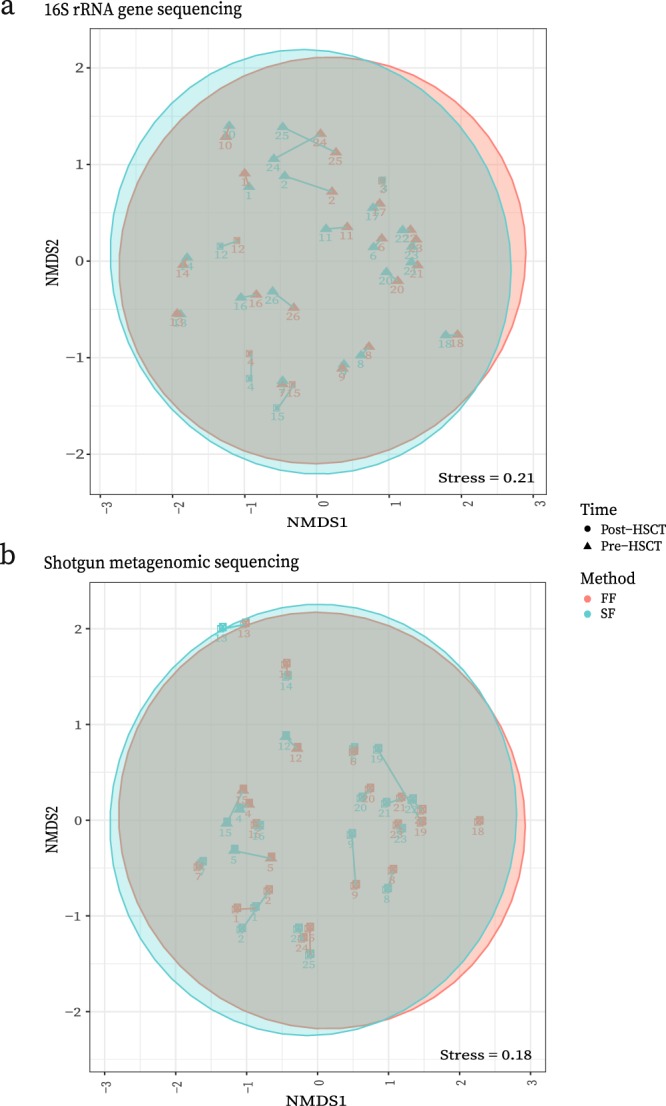
(**a**) 16S rRNA gene sequencing data of beta diversity using Bray-Curtis distances to compare sampling methods represented in a NMDS plot. Sampling pairs are connected by lines with circle vs triangle shapes representing pre-HSCT vs post-HSCT samples respectively. FF = Fresh-frozen, SF = Stabilized-frozen. (**b**) Shotgun metagenomic sequencing data of beta diversity using Bray-Curtis distances to compare sampling methods represented in a NMDS plot. Sampling pairs are connected by lines with circle vs triangle shapes showing sampling time relative to HSCT. FF = Fresh-frozen, SF = Stabilized-frozen. These figures have been created with help of the R(v3.5.0)^29^/PhyloSeq^37^ package.

A non-metric multidimensional scaling (NMDS) plot based on Bray-Curtis distances showed a strong overlap of sampling method types and clustering of sample pairs (Fig. 4a). An ADONIS test on the Bray-Curtis distances showed no evidence of differences between *SF* and *FF* samples (*P* = 1.00).

#### Shotgun metagenomic sequencing data

As with the 16S rRNA gene sequencing data, the NMDS plot showed a strong overlap of sampling methods and clustering of sample pairs (Fig. 4b). The ADONIS again showed no evidence of differences between samples being based on sampling method (*P* = 0.67).

#### Taxonomy and relative abundance

To look more specifically at possible compositional differences, i.e. if certain bacteria were affected by sampling methods, we compared taxonomical differences between *FF* and *SF* samples by calculating the relative abundances of their genera.

#### 16S rRNA gene sequencing data

**Figure 5:**
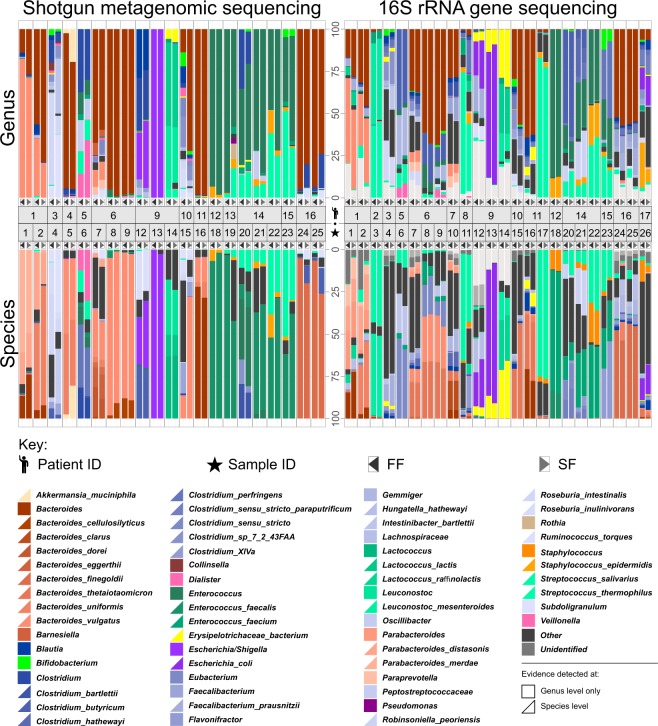
Shotgun metagenomic and16S rRNA gene sequencing data identifying the bacterial genera and species found in the sample cohort and their relative abundances (in percent) present in each individual sample. “Other” are identified bacteria that are not in the top 30 found genera and species respectively across the cohort. “Unidentified” represent bacteria that are among the top 30 genera/species but are unidentifiable at that level. All top 30 bacterial species and genera are represented by a colour shown in the figure key. These colours are displayed as either a square or triangle: (square) evidence found at genus level, colours only map to genus panels; and (triangle) evidence found for species, colours map to both genus and species panels. There are more than 30 species/genera due to the different taxonomies used for 16S rRNA gene and shotgun metagenomic sequencing analysis. This figure has been created with help of the R(v3.5.0)^29^/PhyloSeq^37^ package. For specific values of all relative abundances used in this figure (i.e. the top 30 genera/species from all samples) please see Supplementary Table S2).

Relative abundances of genera between *FF* and *SF* sample pairs appeared visually similar when examining bacteria present in the sample cohort (Fig. 5). This was verified statistically by paired DESeq2 analysis, which found no significant difference (α = 5%) in the relative abundances of all genera between *FF* and *SF* methods.

#### Shotgun metagenomic sequencing

As with the 16S rRNA gene sequencing data, relative abundances of genera were visually similar (Fig. 5**)** but the Wald test of paired DESeq2 highlighted statistically significant differences between *FF* and *SF* in the relative abundance of 7 genera (*Actinomyces*, *Brevibacterium*, *Cryptobacterium*, *Propionibacteriaceae* (unclassified), *Propionibacterium*, *Ralstonia*, *Ruminococcus*, *P* < 0.001 for all). These differences were, however, found in a maximum of 3/21 sample pairs per bacterial genus, with all but one sample pair having differences in abundancy of < 3% (Supplementary Fig. S2). This highlights the sensitivity and reproducibility of shotgun-based sequencing, but also demonstrates the lack of consistent or predictable change one would expect if driven by sampling methodology.

## Discussion

In our study we show that *SF* is comparable to *FF* protocols in a diseased and hospitalized cohort using both 16S rRNA gene and shotgun metagenomic sequencing data. When comparing the alpha-diversity between sampling methods we found no significant difference between *SF* and *FF* samples. Differences between samples (based on Bray-Curtis distances) in our study were not driven by the different sampling methods. Both findings are in agreement with the current literature based on healthy cohorts^5–9,16,22^.

One concern with the use of chemical stabilization was the introduction of biases with regard to changes in the relative abundance of certain bacteria. Two studies using 16S rRNA gene sequencing found a significant increase in the *Sutterella* genus in *SF* versus *FF* samples^5,9^. While another study found significant differences in the relative abundances of *Faecalibacterium*, *Sporobacter*, *Clostridium XVIII*, and *Clostridium XIVa*, with differences being exacerbated in samples of low diversity^8^. However, we found no difference in the relative abundance of these or any other bacteria in our 16S rRNA gene sequencing analysis. In our shotgun metagenomic analysis there were minor differences in several bacteria driven only by a few sample pairs. This shows that differences in taxonomical abundance between methods are inconsistent across studies and may be due to background noise. Additionally, in the case of Hill *et al*., differences could also stem from comparing the *SF* protocol with directly processed samples (i.e. direct DNA extraction prior to freezing). Moreover, previous studies in healthy subjects support our conclusion being that there is little difference between *SF* and *FF* methods^5–7,14–16,18^.

Limitations to our study may include the non-homogenisation of samples. It has been shown that sampling from different parts of a stool can give different microbial compositional results^20^. However, stool sampling is random and therefore the paired design of our study should control for the non-homogenisation. Another potential limitation to our study is the fact that a number of *FF* samples were not frozen directly (Fig. 1c and Supplementary Table S1). However, all of these samples were kept at 4 °C until frozen. Previous studies have shown that cold storage can preserve microbial composition for a short period of time and up to 1 week^6,8,9,26,27^. This difficulty in direct freezing highlights the need for methodologies that allow for more flexibility between sampling and freezing, as immediate freezing is not always possible in a hospital setting. Finally, there was a slight batch effect in our 16S rRNA gene sequencing data due to an uneven distribution of *FF* versus *SF* samples in sequencing runs. This mainly affected reverse-end sequence quality and related variance between batches was minimized during processing. All reads went through the same quality control process, as described in our methods and results, and there were no observed microbial compositional differences between samples run on different batches. Importantly, batch-free shotgun metagenomic data yielded markedly similar results to the 16S rRNA gene sequencing data.

In conclusion, chemical stabilization (*SF*) proved to be comparable to freshly frozen (*FF*) stool samples in a diseased, low microbial diversity, cohort. We confirmed this by statistical examination of measurements by two independent high-throughput sequencing technologies, namely 16S rRNA gene and shotgun metagenomic sequencing. We further showed that use of stabilization techniques maintains reproducibility in cross-sectional studies by minimizing technical variance in sample handling. Our results support the use of chemical stabilization to increase the feasibility of hospital biobank stool sample collection, which will help facilitate the collection of longitudinal faecal microbiome samples from diseased patient groups.

## Methods

### Sample collection

Two samples (i.e. aliquots) were taken from each stool by the patient or nursing staff at the same time-point. The EasySampler® Stool Collection Kit was used to hold the stool above toilet water during collection. If patients were bedridden, samples were collected directly from stool in bedpans. No stool was stored prior to sampling (i.e. sampling was done directly after defecation) and stool material was not homogenised. *FF* samples were placed in a non-sterile tube without stabilization fluid and delivered to the PERSIMUNE biobank by urgent hospital carrier and frozen directly during regular working hours (i.e. Monday-Friday, 8am-3pm). If outside of regular working hours, the *FF* sample would still be delivered to the biobank by urgent hospital carrier but then placed in the biobank refrigerator at 4 °C until regular working hours when it would then be frozen. *SF* samples were placed in the OMNIgene.GUT tube (containing stabilization fluid) according to the manufacturer’s instructions and delivered to the PERSIMUNE biobank by non-urgent hospital carrier. Non-urgent hospital carrier meant that samples could be kept at room temperature for up to several days prior to freezing. Due to the need for swift delivery of *FF* samples, no samples were taken at patients’ homes, i.e. all stool was collected at the hospital.

All samples were handled by personnel at the PERSIMUNE biobank. Half of the *SF* samples (n = 13) were further aliquoted into two sterile tubes prior to freezing, whereas remaining *SF* samples and all *FF* samples were frozen directly without further aliquoting. All samples were frozen at −80 °C.

The OMNIgene.GUT (DNA Stabilized-frozen Inc., Ottawa, Canada) stabilisation tube was chosen as the best candidate for our *SF* protocol. No commercial influence on this decision was allowed, i.e. this decision was not made to promote a specific brand, we did not consult with marketing holders, and did not receive financial support. The decision was based on a review of available documentation and literature: OMNIgene.GUT was found to have the least effect on gut microbiome composition compared to other stabilization fluids^9^, with results generally being comparable to *FF* samples^5,8,15^ and differences among sampling methods being of a lower scale than those between individuals^6,8,16^. The OMNIgene.GUT stabilisation tube should allow samples to be kept at room temperature for up to 60 days (according to manufacturer guidelines) and a previous study (based on a healthy cohort) has shown that the OMNIgene.GUT collection kit gave similar results after 28 days at room temperature as samples with freshly-extracted DNA^7^. For additional information regarding the stabilizer decision process, and an overview of stabilization comparison studies please see **Supplementary Methods**.

### Sample shipment

All samples (26 *FF* and 26 *SF*, i.e. 52 paired samples) were transported on dry ice to the IrsiCaixa AIDS Research Institute in Spain for 16S rRNA gene and shotgun metagenomic sequencing.

### DNA extraction

All samples were thawed on ice and DNA was extracted from ~200 mg of each faecal sample using the PowerSoil DNA Extraction Kit (MO BIO Laboratories, Carlsbad, CA, US). Extracted DNA was then stored at −80 °C until sequencing.

### Library construction and sequencing

#### 16S rRNA gene sequencing

DNA was PCR amplified using primers for targeting regions flanking the variable regions V3-4 of the 16S rRNA gene (expected amplicon size ~460 base pairs). Primers were those described in the “MiSeq rRNA Amplicon Sequencing” protocol from Illumina, which already have the Illumina adapter overhang nucleotide sequences added to the 16S rRNA V3-V4 specific-primers, i.e.: 16S_F 5′-(TCG TCG GCA GCG TCA GAT GTG TAT AAG AGA CAG CCT ACG GGN GGC WGC AG)-3′ and 16S_R 5′-(GTC TCG TGG GCT CGG AGA TGT GTA TAA GAG ACA GGA CTA CHV GGG TAT CTA ATC C)-3′.

Amplifications were performed in triplicate 25 μL reactions, each containing 2.5 μL of non-diluted DNA template, 12.5 μL of KAPA HiFi HotStart Ready Mix (containing KAPA HiFi HotStart DNA Polymerase, buffer, MgCl2, and dNTPs, KAPA Biosystems Inc., Wilmington, MA, USA), and 5 μL of each primer at 1 μM. Thermal cycling conditions consisted of an initial denaturation step (3 min at 95 °C), followed by 30 cycles of denaturation (30 sec at 95 °C), annealing (30 sec at 55 °C) and extension (30 sec at 72 °C). These were followed by a final extension step of 10 min at 72 °C. Once the desired amplicon was confirmed in 1% agarose gel electrophoresis, all three replicates were pooled and stored at −30 °C until sequencing library preparation. Amplified DNA templates were cleaned-up for non-DNA molecules and Illumina sequencing adapters and dual indices were attached using Nextera XT Index Kit (Illumina, Inc.) followed by the corresponding PCR amplification program as described in the MiSeq. 16S rRNA Amplicon Sequencing protocol. After a second round of clean-up, amplicons were quantified using Quant-iT™ PicoGreen® dsDNA Assay Kit (Invitrogen, Carlsbad, MA, USA) and diluted in equimolar concentrations (4 nM) for further pooling. Sequencing was performed on an Illumina MiSeqTM platform according to the manufacturer’s specifications to generate paired-end reads of 300 base-length in each direction. Samples were sequenced in two batches (not all sample pairs were sequenced in the same batch).

#### Shotgun metagenomic sequencing

Whole faecal DNA was chemically fragmented using the Nextera-XT® Illumina kit. One library of approximately 300-basepair-clone insert size was constructed for each sample. Total faecal DNA was sequenced in five runs on an Illumina Hi-Seq® platform at the *Institut de Medicina Predictiva del Cancer* (IMPPC), Badalona, Spain.

### Pre-processing and quality control

#### 16S rRNA gene sequencing data

The FastQC software^28^ and the R(v3.5.0)^29^/DADA2^30^ plotQualityProfile was used to assess raw sequence quality. Primer trimming and read merging was performed using Cutadapt^31^ and PEAR (Illumina Paired-End reAd merger)^32^. Non-merging reads and those with unidentified primers were discarded. Resulting reads were put into the R(v3.5.0)^29^/DADA2^30^ pipeline and subsequently quality filtered and trimmed. Briefly, reads were trimmed by 6 nucleotides from the start of the read, all were truncated at the first nucleotide with a quality score of ≤5 (if there was one) and those with ≥2 expected errors in the read were discarded. Reads with <350 base pairs after filtering and trimming were removed. One million random filtered reads were used to create the error model in DADA2^30^. Chimeric sequences were filtered off using a consensus approach. Samples with <10,000 read counts after processing were discarded. If one sample failed quality control the sample pair was removed from further analysis.

#### Shotgun metagenomic sequencing data

Specific adapters were removed using the Illumina HiSeq software and Trimmomatic^33^. Trimmomatic^33^ was also used for quality trimming (nucleotides were trimmed from the start or end of the read if they had a quality score <30, then using a sliding window approach, reads were cut if the average quality of 4 nucleotides was <30). Reads <50 base pairs were removed. Paired reads were mapped with bowtie2^34^ to the human genome (GRCh38). All read pairs where one or both mates mapped to the human genome were discarded. Final read quality was assessed using the R package ShortRead^35^ and bowtie2^34^ output files. Samples with <1 million reads were removed from further analysis. If one sample failed quality control the sample pair was removed from further analysis.

### Ethical approval

This study was conducted with approval from the Danish Ethical Committee (J.nr.H-16047481). All patients had given informed consent to the donation and storage of stool samples for microbial research. All methods were performed in accordance with the relevant guidelines and regulations.

### Taxonomy and diversity measures

#### 16S rRNA gene sequencing data

Taxonomy was assigned through alignment to the RDP (Ribosomal Database Project)^36^ using the R(v3.5.0)^29^/DADA2^30^ program. Taxonomical annotation was used to remove all OTUs assigned as mitochondria or chloroplasts, all OTUs assigned a non-bacterial kingdom and all OTUs present in <2 samples. The filtered OTU table was used in the R(v3.5.0)^29^/Phyloseq^37^ package to calculate relative abundances (at genus and species level), alpha diversity measures (Shannon and Simpson indexes) and beta diversity measures (Bray-Curtis dissimilarity; used as input for ordination analysis using non-metric multidimensional scaling (NMDS)). Specifically, relative abundances were based on OTU counts and were normalised as percentages (100 * (x/sum(x))) at species and genera taxonomy levels. Alpha diversity measures were calculated based on OTU counts without prior normalisation. Beta diversity measures were calculated based on normalised (x/sum(x)) OTU counts.

#### Shotgun metagenomic sequencing data

Taxonomical annotation was assigned using MetaPhlAn v2.0^38^. The relative proportions calculated from MetaPhlAn v2.0^38^ on genus and/or species level were used to calculate relative abundances, alpha diversity measures (Shannon and Simpson indexes) and beta diversity measures (Bray-Curtis dissimilarity; used as input for ordination analysis using non-metric multidimensional scaling (NMDS)) in the R(v3.5.0)^29^/Phyloseq^37^ package. Relative abundances were calculated as percentages (i.e. relative proportions were multiplied by 100) at both species and genera taxonomy levels. Due to alpha diversity measures often being calculated based on unnormalized data, we used the rounded fraction of the relative proportions of bacterial species from MetaPhlAn v2.0^38^ (i.e. relative proportion * 10,000 rounded up to the closest integer) to calculate these measures. For beta diversity measures the relative proportion values of bacterial species could be used directly (i.e. values did not need to be re-normalised prior to calculation).

MetaPhlAn v2.0^38^ also provided estimations of the number of reads that should come from a given species by considering the coverage of the species’ markers and the length of the genome (taken from reference genomes). Using the estimated number of reads per genera a count table was created for shotgun taxonomical data compatible with the R(v3.5.0)^29^/PhyloSeq^37^ package. This count table, based on the estimation of reads from bacterial genera, was used for later DESeq2^39^ analyses.

Gene richness was calculated using bowtie2^34^ to map filtered reads to the IGC (Integrated Gene Catalogue)^25^. Based on a rarefaction curve of number of new unique genes and number of mapped reads (counting forward reads only), a cut-off of 5 million mapped reads was decided to calculate gene richness (Supplementary Fig. S1). Only forward reads were used to calculate the number of unique genes, with a maximum of 1 unique gene per read.

### Statistical analysis

The following statistical analyses were performed for both 16S rRNA gene and shotgun metagenomic sequencing data. Detection of differentially abundant bacterial genera between methods was performed using the R(v3.5.0)^29^/PhyloSeq^37^ and R(v3.5.0)^29^/DESeq2^39^ packages. Using the Wald test in DESeq2^39^, *SF* and *FF* methods were compared for potential statistical differences in their geometric means of bacterial genera. PERMANOVA (adonis) tests (via the R(v3.5.0)^29^/vegan package^40^) were also performed using Bray-Curtis distances to test for a potential association between sampling methods and microbiota composition. For all statistical analyses, *P*-values < 0.05 were considered significant.

#### 16S rRNA gene sequencing data

The filtered OTU count table created through R(v3.5.0)^29^/DADA2^30^ with taxonomy down to genera-level was used for the DESeq2^39^ analysis. Normalised (x/ (sum (x)) OTU values from the same table were used for the PERMANOVA (adonis) test.

#### Shotgun metagenomic sequencing data

Values from the created count table (based on the estimated number of reads from bacterial genera calculated by MetaPhlAn v2.0^38^) was used for the DESeq2^39^ analysis. For the PERMANOVA (adonis) test, proportional abundances (calculated by MetaPhlAn v2.0^38^) were used. Gene richness differences between methods (shotgun metagenomic sequencing data only) were assessed using the Wilcoxon signed-rank test.

## Supplementary information

Supplementary Information

Supplementary Table S2

## Data Availability

The datasets generated and analysed during this study are derived from patients treated in Denmark. The datasets contain sensitive patient data governed by GDPR and Danish law. Due to Danish legislation (Act No. 502 of 23 May 2018) and approvals granted by the Danish Data Protection Agency, it is not possible to upload raw data to a publicly available database. However, access to these data can be made available from the corresponding author on reasonable request, provided a data transfer agreement is entered into according to current regulations.
