# Supplementary material for: Gut microbiome comparability of fresh-frozen versus stabilized-frozen samples from hospitalized patients using 16S rRNA gene and shotgun metagenomic sequencing
Source: Sci Rep. 2019 Sep 16;9:13351. doi: 10.1038/s41598-019-49956-7 (PMC6746779; doi:10.1038/s41598-019-49956-7)
Supplement: Supplementary file 1 — Supplementary Information [file 41598_2019_49956_MOESM1_ESM.pdf]

Supplementary Information

Article Title

Gut microbiome comparability of fresh-frozen versus stabilized-frozen samples from hospitalized patients using 16S rRNA gene and shotgun metagenomic sequencing

Authors

Emma E. Ilett[1*], Mette Jørgensen[1], Marc Noguera Julian[2], Gedske Daugaard[3], Daniel D. Murray[1], Marie Helleberg[1], Roger Paredes[2,4], Jens Lundgren[1], Henrik Sengeløv[5**], Cameron MacPherson[1**]

*Correspondence: emma.elizabeth.ilett.01@regionh.dk (Phone: +45 35 45 58 88)

**: shared senior authorship

[1]PERSIMUNE Centre of Excellence, Rigshospitalet, Blegdamsvej 9, DK-2100, Copenhagen Ø, Denmark. [2]Institut de Recerca de la SIDA – IrsiCaixa, Hospital Universitari Germans Trias i Pujol, Badalona, Catalonia, Spain. [3]Department of Oncology, Rigshospitalet Blegdamsvej 9, DK-2100, Copenhagen Ø, Denmark. [4]Infectious Diseases Service, Hospital Universitari Germans Trias i Pujol, Badalona, Spain. [5]Department of Haematology, Rigshospitalet, Blegdamsvej 9, DK-2100, Copenhagen Ø, Denmark.

Supplementary Table S1

Patient	Sample	Method	HSCT type	HSCT to sampling (days)	Sampling to freezing (days)
1	1	*FF*	Myeloablative HSCT	7	0
1	1	*SF*	Myeloablative HSCT	7	0
1	2	*FF*	Myeloablative HSCT	14	NA[1]
1	2	*SF*	Myeloablative HSCT	14	NA[1]
2	3	*FF*	Myeloablative HSCT	7	0
2	3	*SF*	Myeloablative HSCT	7	9
3	4	*FF*	Myeloablative HSCT	-28	0
3	4	*SF*	Myeloablative HSCT	-28	0
4	5	*FF*	Non-myeloablative HSCT	-150	2
4	5	*SF*	Non-myeloablative HSCT	-150	2
5	6	*FF*	Non-myeloablative HSCT	28	7
5	6	*SF*	Non-myeloablative HSCT	28	7
6	7	*FF*	Myeloablative HSCT	-22	2
6	7	*SF*	Myeloablative HSCT	-22	2
6	8	*FF*	Myeloablative HSCT	14	0
6	8	*SF*	Myeloablative HSCT	14	0
6	9	*FF*	Myeloablative HSCT	21	0
6	9	*SF*	Myeloablative HSCT	21	0
7	10	*FF*	Myeloablative HSCT	-19	7
7	10	*SF*	Myeloablative HSCT	-19	7
8	11	*FF*	Non-myeloablative HSCT	10	1
8	11	*SF*	Non-myeloablative HSCT	10	1
9	12	*FF*	Non-myeloablative HSCT	-28	0
9	12	*SF*	Non-myeloablative HSCT	-28	0
9	13	*FF*	Non-myeloablative HSCT	20	0
9	13	*SF*	Non-myeloablative HSCT	20	13
9	14	*FF*	Non-myeloablative HSCT	26	0
9	14	*SF*	Non-myeloablative HSCT	26	7

10	15	*FF*	Myeloablative HSCT	-29	0
10	15	*SF*	Myeloablative HSCT	-29	0
11	16	*FF*	Non-myeloablative HSCT	7	0
11	16	*SF*	Non-myeloablative HSCT	7	0
11	17	*FF*	Non-myeloablative HSCT	14	0
11	17	*SF*	Non-myeloablative HSCT	14	7
12	18	*FF*	Myeloablative HSCT	21	1
12	18	*SF*	Myeloablative HSCT	21	8
13	19	*FF*	Myeloablative HSCT	14	0
13	19	*SF*	Myeloablative HSCT	14	8
14	20	*FF*	Myeloablative HSCT	14	0
14	20	*SF*	Myeloablative HSCT	14	2
14	21	*FF*	Myeloablative HSCT	21	0
14	21	*SF*	Myeloablative HSCT	21	9
14	22	*FF*	Myeloablative HSCT	29	0
14	22	*SF*	Myeloablative HSCT	29	14
15	23	*FF*	Myeloablative HSCT	14	3
15	23	*SF*	Myeloablative HSCT	14	16
16	24	*FF*	Myeloablative HSCT	14	3
16	24	*SF*	Myeloablative HSCT	14	7
16	25	*FF*	Myeloablative HSCT	21	0
16	25	*SF*	Myeloablative HSCT	21	8
17	26	*FF*	Myeloablative HSCT	14	1
17	26	*SF*	Myeloablative HSCT	14	14

Baseline table of patients, samples, sampling time points and time to freezing. *FF*= Fresh-frozen, *SF*= Stabilized-frozen.

[1]NA = the nurse/patient taking the sample had not noted the time of sampling. The sampling to freezing value for this sample is therefore marked as NA for both the SF and FF sample from the same stool.

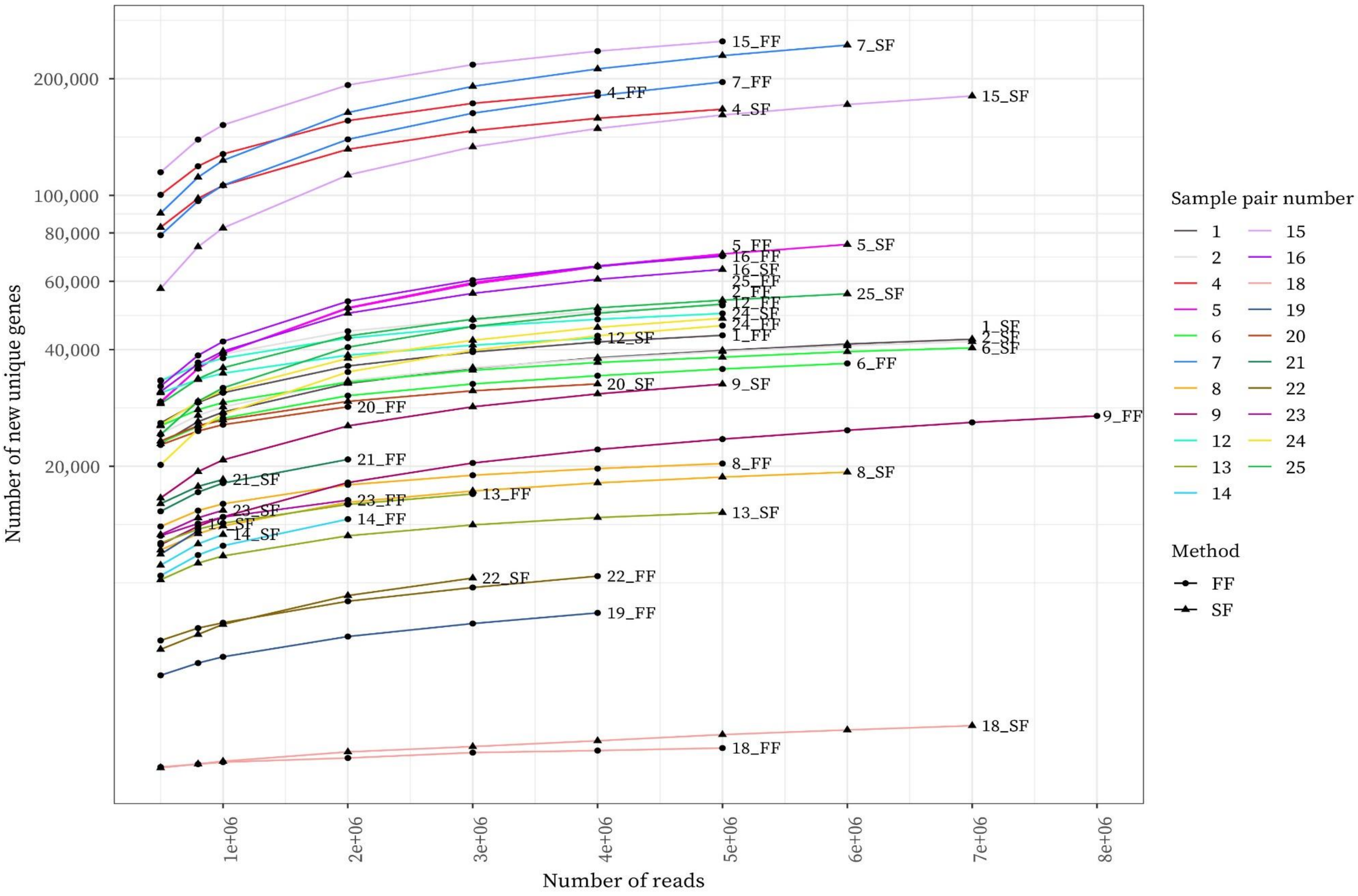
Number of new unique genes
200,000
100,000
80,000
60,000
40,000
20,000
Number of reads
1e+06
2e+06
3e+06
4e+06
5e+06
6e+06
7e+06
8e+06
15_FF
7_SF
7_FF
4_FF
4_SF
15_SF
5_FF
16_FF
16_SF
5_SF
25_FF
2_FF
12_FF
24_SF
24_FF
1_FF
25_SF
1_SF
2_SF
6_SF
12_SF
6_FF
20_SF
9_SF
20_FF
9_FF
21_FF
21_SF
8_FF
8_SF
23_FF
13_FF
23_SF
14_FF
13_SF
14_SF
22_SF
22_FF
19_FF
18_SF
18_FF
Sample pair number
1
2
4
5
6
7
8
9
12
13
14
15
16
18
19
20
21
22
23
24
25
Method
FF
SF

Supplementary Figure S1. Gene richness rarefaction curve per sample and method. Each individual sample per sample pair is labelled (sample pair number_sampling method, e.g. 1_SF (sample pair 1, sampling method= stabilized-frozen)). There is a saturation of new unique genes for the majority of samples after the 5 million reads mark. Based on forward reads mapping to the IGC (Integrated Gene Catalogue) catalogue. FF= Fresh-frozen, SF= Stabilized-frozen.

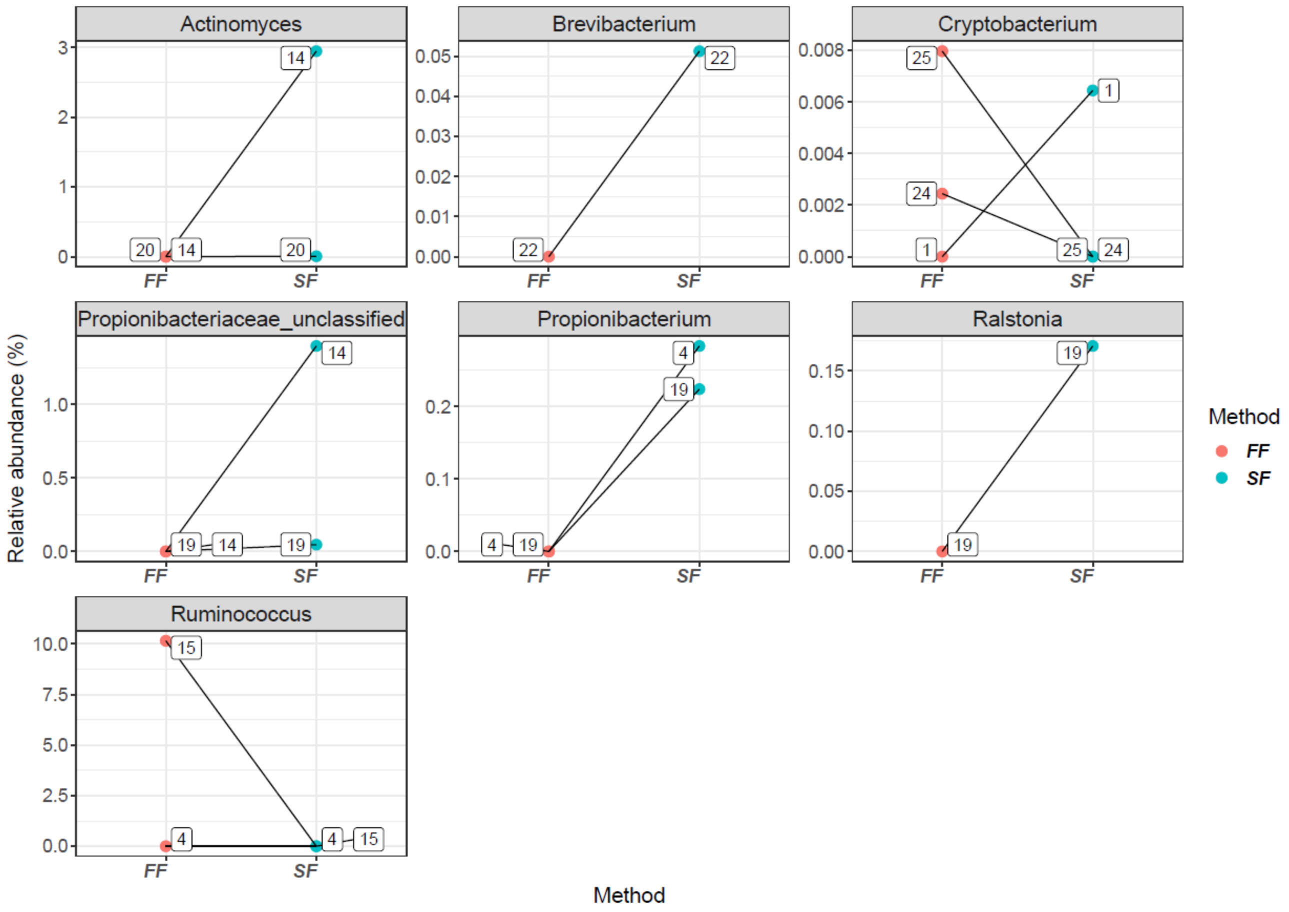

Supplementary Figure S2. The paired DESEQ2 analysis on shotgun metagenomic data showed significant differences in the abundance of seven bacterial genera. Here we show the sample pairs that had these differences for each bacterial genera. *FF*= Fresh-frozen, *SF*= Stabilized-frozen.

Supplementary Methods

Stabilizer choice:

Several stabilizers are available for the sampling of stool for later microbiome analysis and claim to allow for a delay prior to freezing (*SF* (stabilized-frozen) sampling). The main commercially available stabilization fluids include RNAlater, 70% or 95% ethanol, guanidine, PSP buffer and OMNIgene.GUT. Gut microbiome studies have also been performed using stool from swabs or FTA (Flinders Technology Associates)/FOBT (faecal occult blood test)/FIT (faecal immunochemical test) cards. A discussion of these additional sampling methods is beyond the scope of this article.

Among studies assessing different *SF* collection methods (all in non-hospitalized/healthy cohorts), most have found fresh-frozen (*FF*) and *SF* sampling methods to be comparable[1,2]. However, several studies have found not to recommend 70% ethanol[3,4], and others have found RNAlater to give a lower DNA yield and lower bacterial diversity compared to *FF* samples[5-8], as well as a loss in stability when at ambient temperatures for a longer period of time[3,9]. Studies evaluating the OMNIgene.GUT. (DNA Genotek Inc.) stabilization fluid have found it to have the least effect on gut microbiome composition compared to other *SF* methods[3,7,10,11]. Importantly, the OMNIgene.GUT stabilization fluid has been shown to give similar results after 28 days at room temperature as samples with freshly extracted DNA (*FE*) samples[12]. Some have found minor differences with the OMNIgene.GUT tube with regard to bacterial abundance proportions[7,13-15], but results have been generally comparable to *FF* sampling[14,16,17], with differences among sampling methods being of a lower scale than those between individuals[13,16]. Thus, our literature review concluded that the OMNIgene.GUT tube should be our *SF* sampling method of choice and should be tested against *FF* samples in our hospital cohort. We cannot make any conclusions regarding other *SF* reagents that were not tested. Again, the company DNA Genotek Inc. played no part in this review, and all sampling kits were financed without aid from the company. No authors or hospital staff have received financial support or have any stockholdings in DNA Genotek Inc.

Overview of human stool sample collection/stabilizer comparison studies from 2010 onwards

Reference	Year	Journal	N subjects	Subject type	*FF*	*FE*	*SF*	16S rRNA gene sequencing (region)	Shotgun metagenomic sequencing	Sequencing machine
**Wu et al.[1]**	2010	BMC Microbiology	10	Adults	Yes	Yes	PSP (Invitek) buffer	Yes (V1-V2)	No	454 FLX machine Roche
**Dominianni et al.[5]**	2013	BMC Microbiology	3	Adults	Yes	No	RNAlater	Yes (V3-V4)	No	454 FLX machine Roche
**Choo et al.[7]**	2015	Scientific reports	1	Adults	Yes	No	OMNIgene.GUT, RNAlater, Tris-EDTA	Yes (V4)	No	Illumina MiSeq
**Flores et al.[18]**	2015	Microbiome	10	Adults	Yes	No	RNAlater, RNAlater+ kanamycin, RNAlater+ ciprofloxacin	Yes (V3-V4)	No	Illumina MiSeq
**Gorzelak et al.[6]**	2015	PLOSone	4	Adults	Yes	No	RNAlater	Yes (target bacterial groups)	No	Biorad CFX 96 real time PCR detection system
**Mathy et al.[11]**	2015	Biopreservation and Biobanking	3	Infants (+3 dogs)	Yes	No	OMNIgene.GUT (P-084 and P-085 prototype), PSP Spin Stool DNA plus kit, RNAlater	Yes (V4)	No	Illumina MiSeq
**Voigt et al.[2]**	2015	GenomeBiology	8	Adults	Yes	No	RNAlater	No	Yes	Illumina HiSeq
**Anderson et al.[12]**	2016	Scientific reports	16	Adults	Yes	Yes	OMNIgene.GUT	No	Yes	Illumina HiSeq

**Hill et al.**[16]	2016	Microbiome	44	Infants and older adults	Yes	Yes	OMNIgene.GUT	Yes (V4-V5)	No	Illumina MiSeq
**Song et al.**[3]	2016	mSystems	10	Adults (+ 5 dogs)	Yes	Yes	OMNIgene.GUT, 70% ethanol, 95% ethanol, Flinders Technology Associates (FTA) cards	Yes (V4)	No	Illumina HiSeq and Illumina MiSeq
**Al et al.**[19]	2017	Journal of Microbiological Methods	3	Adults	Yes	Yes	RNAlater	Yes (V4)	No	Illumina MiSeq
**Vogtmann et al.**[9]	2017	American Journal of Epidemiology	52	Adults	Yes	No	95% ethanol, RNAlater, faecal occult blood test cards, faecal immunochemical test tubes	Universal primer set 515F/806R	No	Illumina HiSeq
**Angebault et al.**[8]	2018	PLOSone	3	Adults	Yes	No	RNAlater	Yes (V3-V4) (+ITS)	No	454 FLX machine Roche
**Han et al.**[15]	2018	Microbiome	8	Adults	Yes	Yes	OMNIgene.GUT, Novel NOBp-based	No	Yes	Illumina HiSeq + BGISEQ-500
**Panek et al.**[14]	2018	Scientific reports	4	Adults	Yes	Yes	OMNIgene.GUT	Yes (V3-V4 for Illumina, V2, V4, V8 and V3, V6-V7, V9 for Ion Torrent)	No	Illumina MiSeq + Ion Torrent PGM

**Penington et al.**[13]	2018	Scientific reports	6	Adults	Yes	No	OMNIgene.GUT	Yes (V4)	No	Illumina MiSeq
**Ribeiro et al.**[20]	2018	Journal of the Sao Paulo Institute of Tropical Medicine	10	Adults	Yes	Yes	Guanidine	Yes (V4)	No	Ion Torrent PGM
**Szopinska et al.**[17]	2018	BMC Microbiology	14	Adults	No	Yes	OMNIgene.GUT	Yee (V3-V4)	No	Illumina MiSeq
**Wang et al.**[21]	2018	Frontiers in Cellular and Infection Microbiology	8	Healthy	Yes	No	OMNIgene.GUT, 95% ethanol, RNAlater, Flinders Technology Associates (FTA) cards	Yes (V4)	No	Illumina MiSeq
**Ezzy et al.**[22]	2019	Journal of Microbiological Methods	3	Healthy	Yes	No	DETA (20% DMSO-0.25M EDTA, pH 8.0), DETA-NaCl, 95% ethanol*	Yes (V3-V4)	No	Illumina MiSeq

*Only samples stored with DETA-NaCl as well as fresh-frozen, -20ºC and -80ºC storage samples were 16S rRNA gene sequenced (other methods were assessed for DNA yield and purity only)

References

1 Wu, G. D. *et al.* Sampling and pyrosequencing methods for characterizing bacterial communities in the human gut using 16S sequence tags. *BMC microbiology* **10**, 206, doi:10.1186/1471-2180-10-206 (2010).

2 Voigt, A. Y. *et al.* Temporal and technical variability of human gut metagenomes. *Genome biology* **16**, 73, doi:10.1186/s13059-015-0639-8 (2015).

3 Song, S. J. *et al.* Preservation Methods Differ in Fecal Microbiome Stability, Affecting Suitability for Field Studies. *mSystems* **1**, doi:10.1128/mSystems.00021-16 (2016).

4 Sinha, R. *et al.* Collecting Fecal Samples for Microbiome Analyses in Epidemiology Studies. *Cancer epidemiology, biomarkers & prevention : a publication of the American Association for Cancer Research, cosponsored by the American Society of Preventive Oncology* **25**, 407-416, doi:10.1158/1055-9965.epi-15-0951 (2016).

5 Dominianni, C., Wu, J., Hayes, R. B. & Ahn, J. Comparison of methods for fecal microbiome biospecimen collection. *BMC microbiology* **14**, 103, doi:10.1186/1471-2180-14-103 (2014).

6 Gorzelak, M. A. *et al.* Methods for Improving Human Gut Microbiome Data by Reducing Variability through Sample Processing and Storage of Stool. *PloS one* **10**, e0134802, doi:10.1371/journal.pone.0134802 (2015).

7 Choo, J. M., Leong, L. E. & Rogers, G. B. Sample storage conditions significantly influence faecal microbiome profiles. *Scientific reports* **5**, 16350, doi:10.1038/srep16350 (2015).

8 Angebault, C. *et al.* Combined bacterial and fungal intestinal microbiota analyses: Impact of storage conditions and DNA extraction protocols. *PloS one* **13**, e0201174, doi:10.1371/journal.pone.0201174 (2018).

9 Vogtmann, E. *et al.* Comparison of Collection Methods for Fecal Samples in Microbiome Studies. *American journal of epidemiology* **185**, 115-123, doi:10.1093/aje/kww177 (2017).

10 Mehta, R. S. *et al.* Stability of the human faecal microbiome in a cohort of adult men. *Nature microbiology* **3**, 347-355, doi:10.1038/s41564-017-0096-0 (2018).

11 Mathay, C. *et al.* Method optimization for fecal sample collection and fecal DNA extraction. *Biopreservation and biobanking* **13**, 79-93, doi:10.1089/bio.2014.0031 (2015).

12 Anderson, E. L. *et al.* A robust ambient temperature collection and stabilization strategy: Enabling worldwide functional studies of the human microbiome. *Scientific reports* **6**, 31731, doi:10.1038/srep31731 (2016).

13 Penington, J. S. *et al.* Influence of fecal collection conditions and 16S rRNA gene sequencing at two centers on human gut microbiota analysis. *Scientific reports* **8**, 4386, doi:10.1038/s41598-018-22491-7 (2018).

14 Panek, M. *et al.* Methodology challenges in studying human gut microbiota - effects of collection, storage, DNA extraction and next generation sequencing technologies. *Scientific reports* **8**, 5143, doi:10.1038/s41598-018-23296-4 (2018).

15 Han, M. *et al.* A novel affordable reagent for room temperature storage and transport of fecal samples for metagenomic analyses. *Microbiome* **6**, 43, doi:10.1186/s40168-018-0429-0 (2018).

16 Hill, C. J. *et al.* Effect of room temperature transport vials on DNA quality and phylogenetic composition of faecal microbiota of elderly adults and infants. *Microbiome* **4**, 19, doi:10.1186/s40168-016-0164-3 (2016).

17 Szopinska, J. W. *et al.* Reliability of a participant-friendly fecal collection method for microbiome analyses: a step towards large sample size investigation. *BMC microbiology* **18**, 110, doi:10.1186/s12866-018-1249-x (2018).

18 Flores, R. *et al.* Collection media and delayed freezing effects on microbial composition of human stool. *Microbiome* **3**, 33, doi:10.1186/s40168-015-0092-7 (2015).

19 Al, K. F., Bisanz, J. E., Gloor, G. B., Reid, G. & Burton, J. P. Evaluation of sampling and storage procedures on preserving the community structure of stool microbiota: A simple at-home toilet-paper collection method. *Journal of microbiological methods* **144**, 117-121, doi:10.1016/j.mimet.2017.11.014 (2018).

20 Ribeiro, R. M. *et al.* An alternative storage method for characterization of the intestinal microbiota through next generation sequencing. *Revista do Instituto de Medicina Tropical de Sao Paulo* **60**, e77, doi:10.1590/s1678-9946201860077 (2018).

21 Wang, Z. *et al.* Comparison of Fecal Collection Methods for Microbiome and Metabolomics Studies. *Frontiers in cellular and infection microbiology* **8**, 301, doi:10.3389/fcimb.2018.00301 (2018).

22 Ezzy, A. C. *et al.* Storage and handling of human faecal samples affect the gut microbiome composition: A feasibility study. *Journal of microbiological methods* **164**, 105668, doi:10.1016/j.mimet.2019.105668 (2019).
